# Monitoring Inputs, Control Architectures, and Failure Modes in Closed-Loop Vasopressor Systems: A Comprehensive Review

**DOI:** 10.3390/s26072180

**Published:** 2026-04-01

**Authors:** Vitor Felippe, Hiorrana Sousa Dias, Carlos Darcy Alves Bersot, Gustavo Guimaraes Torres, Bruno Wegner, Gabriel Lemos González, Gustavo Wegner, Marcos Adriano Lessa

**Affiliations:** 1 Department of Anesthesiology, National Cancer Institute (INCA), Rio de Janeiro 20230-130, RJ, Brazil; vitorfelippe2@gmail.com; 2 School of Medicine, Centro Universitário Christus (UNICHRISTUS), Fortaleza 60192-345, CE, Brazil; hiorrana.dias@hotmail.com; 3 Department of Anesthesiology, Santa Casa de Misericórdia de Campos dos Goytacazes, Campos dos Goytacazes 28030-002, RJ, Brazil; carlosbersot@gmail.com; 4 Postgraduate Program in Translational Medicine, Paulista School of Medicine (EPM-UNIFESP), São Paulo 04023-062, SP, Brazil; 5 Faculty of Medical Sciences, Hospital Universitário Pedro Ernesto, State University of Rio de Janeiro (UERJ), Rio de Janeiro 20551-030, RJ, Brazil; 6 Faculty of Medicine, Federal University of Rio Grande do Sul (UFRGS), Porto Alegre 90610-264, RS, Brazil; 7 School of Medicine and Surgery, Federal University of the State of Rio de Janeiro (UNIRIO), Rio de Janeiro 20270-330, RJ, Brazil; gonzalez.anest@gmail.com; 8 Faculty of Medicine, Federal University of Fronteira Sul (UFFS), Passo Fundo 99010-200, RS, Brazil; gustavoroberto5@hotmail.com; 9 Department of Anesthesia, Carver College of Medicine, University of Iowa, Iowa City, IA 52242, USA

**Keywords:** closed-loop control, vasopressor, automation, hemodynamic monitoring, signal integrity, reliability, failure modes, perioperative technology

## Abstract

Closed-loop vasopressor systems integrate real-time blood pressure monitoring with automated decision logic to support hemodynamic stability in perioperative and critical care environments. These technologies sit at the intersection of biomedical sensing, signal processing, and clinician-supervised automation: the quality, latency, and failure behavior of the monitoring input can directly shape controller performance, safety margins, and clinical usability. In this comprehensive review, we synthesize the major closed-loop vasopressor architectures reported in the literature, examine how sensor modality and signal integrity influence algorithm behavior, and summarize recurrent reliability vulnerabilities spanning sensors, control logic, and device integration. We organize the field through an end-to-end information pipeline—monitoring input, signal conditioning and quality assessment, decision and control strategy, actuation via infusion technology, and supervisory safety layers—highlighting common performance metrics used to benchmark control quality. We then discuss clinical validation patterns across settings, emphasizing practical considerations for deployment and the evidence gaps that remain most relevant to high-risk populations. Finally, we propose reporting and validation priorities for future studies, with a focus on sensor robustness, transparency of algorithm design, integration safeguards, and standardized documentation of failures and overrides.

## 1. Introduction

Hemodynamic instability during major surgery and critical illness requires continuous vasopressor titration to maintain adequate tissue perfusion [1,2,3,4]. Manual titration, the current standard of care, is reactive and labor-intensive, with clinician response times typically exceeding two minutes from hypotension onset [4,5]. This delay contributes to cumulative hypotensive exposure, associated with adverse outcomes including organ injury, surgical complications, and increased mortality [2,6]. The cognitive load of continuous blood pressure monitoring and frequent dose adjustments competes with other perioperative tasks, potentially limiting achievable precision. Indeed, observational data indicate that norepinephrine infusions in the ICU and operating room are outside predetermined target ranges approximately 50% of treatment time, illustrating the magnitude of the gap between current practice and optimal hemodynamic control. [7]

Automated hemodynamic control has evolved substantially since early implementations in hypertensive crisis management during the 1970s [8,9]. Initial nitroprusside-based systems demonstrated technical feasibility but remained confined to specialized settings due to sensor limitations, computational constraints, and algorithmic simplicity [9,10]. Over subsequent decades, parallel advances in monitoring technology (continuous noninvasive arterial pressure devices, high-fidelity invasive transducers) and control theory (proportional controllers, proportional–integral–derivative (PID) algorithms, multi-agent rule-based systems) enabled progressive expansion from controlled experimental protocols into broader perioperative applications [8,9,10,11,12]. Contemporary platforms now span low-risk obstetric anesthesia to high-acuity surgical and critical care contexts, reflecting both technological maturation and diversification of clinical targets [11,12,13,14].

Contemporary closed-loop vasopressor systems integrate continuous blood pressure sensors with algorithmic control strategies to automate drug titration [8,10,11]. Monitoring modalities range from intermittent oscillometric measurements to continuous noninvasive finger-cuff devices (e.g., the Edwards Lifesciences Nexfin/ClearSight system, which uses continuous noninvasive arterial pressure [CNAP] estimation via a volume-clamp finger cuff) and invasive arterial catheters, each offering distinct trade-offs between accessibility and signal fidelity [3,15]. Control algorithms span simple on–off threshold logic, proportional controllers, sophisticated proportional–integral–derivative architectures, and complex rule-based systems managing multiple vasopressors simultaneously [8,9,10]. Systems are deployed across diverse settings including the operating room (OR), intensive care unit (ICU), and obstetric suite, targeting mean arterial pressure (MAP), systolic blood pressure (SBP), or hybrid composite endpoints. Performance metrics commonly reported include median absolute performance error (MDAPE) and wobble, which quantify control precision and oscillation, respectively. Heart rate (HR) is an ancillary input in rule-based dual-vasopressor platforms. By eliminating human reaction time and enabling sub-second to one-minute control loops depending on sensor technology, these platforms theoretically improve hemodynamic precision while reducing clinician workload [10,13,15]. Despite four decades of iterative development, comparative performance across diverse clinical contexts, the critical interplay between sensor reliability and algorithmic sophistication, and technical vulnerabilities that limit clinical deployment remain incompletely characterized [9,10].

This comprehensive review addresses key gaps in the translational pathway from closed-loop concept to bedside deployment. Our primary objective is to characterize technological architectures, sensor–algorithm interactions, and system vulnerabilities across diverse clinical applications of closed-loop vasopressor titration. A secondary objective is to summarize how clinical validation studies have assessed performance and outcomes, and to identify priorities for future evaluation and reporting.

## 2. Literature Review Approach

This review focuses on closed-loop systems that titrate vasopressors to a blood pressure target using automated logic driven by physiologic monitoring inputs. We emphasize (i) monitoring modalities and signal characteristics relevant to automated titration, (ii) controller and decision-logic architectures, (iii) integration and actuation constraints (e.g., infusion pump interfaces and update cycles), and (iv) reliability vulnerabilities and safety hazards observed or anticipated in clinical use. Sources were identified through targeted searching of major biomedical databases and iterative citation-chaining of key clinical and technical publications through March 2025. The synthesis is narrative and architecture-centered, prioritizing concepts and failure mechanisms that generalize across devices and clinical contexts.

## 3. Conceptual Framework: The Sensor-to-Actuation Information Pipeline

**Figure 1:**
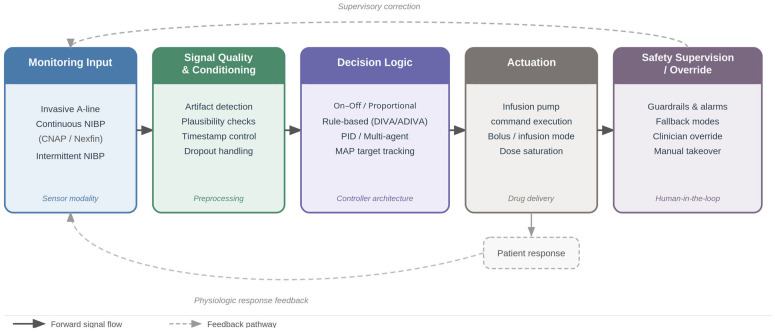
Conceptual schematic of the end-to-end workflow in automated vasopressor titration: monitoring input (invasive arterial line, continuous or intermittent noninvasive blood pressure) → signal quality and conditioning (artifact detection, filtering/validation, latency assessment) → decision logic (e.g., rule-based or PID control with MAP target tracking and dosing constraints) → actuation (infusion pump command execution) → safety supervision/override (guardrails, alarms, fallback behavior, clinician override). Curved feedback pathways depict supervisory correction of controller behavior and physiologic response feedback to subsequent monitoring.

The fundamental premise of this framework is simple: controller performance is bounded by input signal quality. No algorithm, however sophisticated, can compensate for unreliable sensor data; the choice of monitoring technology directly constrains achievable control precision [9,10]. The overall structure of the sensor-to-actuation information pipeline is illustrated in Figure 1.

The effectiveness of closed-loop systems depends on the integrity of the entire information processing chain. Signal acquisition begins with sensor measurement: oscillometric devices [16,17,18] sampled at 1 min intervals, introducing inherent dead-time; CNAP and Nexfin [17,18,19] provided beat-to-beat continuous data but required artifact filtering to remove motion-induced noise; invasive arterial lines [20,21,22,23] delivered high-fidelity continuous waveforms with minimal latency. Signal processing and control computation varied by algorithm: on–off controllers required simple threshold comparison (<1 ms computation); proportional controllers calculated linear scaling (Rate = f(error)); PID systems solved three-term equations incorporating historical error integration and derivative estimation, with control cycles ranging from 1 s to 1 min. Dose delivery involved communication between controller software and infusion pumps, where failures were documented in two studies [24,25]. Patient response completed the loop, with pharmacodynamic delays from drug administration to hemodynamic effect ranging from 1 to 3 min for phenylephrine boluses to 3–5 min for norepinephrine infusion adjustments. The total loop latency from pressure change detection to therapeutic effect ranged from 2 to 4 min in oscillometric systems to 10–30 s in invasive PID systems, fundamentally constraining the achievable control bandwidth [17,18,19,20].

## 4. Monitoring Inputs for Closed-Loop Vasopressor Control

The blood pressure input is not only a measurement source; in closed-loop vasopressor systems it defines the practical limits of control performance. In perioperative and critical care settings, the monitor determines temporal resolution, noise profile, dropout behavior, and calibration stability, all of which shape controller responsiveness and safety margins [3,10,15]. For this reason, discussion of algorithm performance should always be interpreted in the context of input modality rather than in isolation. A highly tuned controller cannot compensate for persistent signal sparsity, recurrent dropout, or unrecognized drift [10,11].

Across available studies, three monitoring strategies dominate: intermittent oscillometric NIBP, continuous noninvasive finger-cuff systems (CNAP/Nexfin/ClearSight), and invasive arterial pressure monitoring [16,17,18,19,20,21,23,25,26,27,28,29,30]. These modalities differ not only in technical fidelity, but also in the type of failure they introduce into the loop. Intermittent NIBP primarily imposes deterministic sampling gaps; finger-cuff systems offer continuity but introduce calibration interruptions and perfusion-sensitive artifacts; invasive arterial lines deliver the highest signal continuity but remain dependent on transducer setup quality and invasive access workflows. Table 1 summarizes these modality-specific constraints and recommended safeguards [9,10,14].

### 4.1. Intermittent Noninvasive Blood Pressure (Nibp)

Intermittent oscillometric NIBP was central to early obstetric implementations and remains attractive because of universal availability, low setup burden, and familiarity in routine anesthesia workflows [18,19,30]. Its key limitation is structural: the loop receives new information only at cuff cycles, creating dead-time between measurements. During this interval, hypotension can emerge and progress without direct feedback, particularly during rapid sympathetic shifts after neuraxial blockade or abrupt surgical stimulation changes [18,30].

This sparsity has two practical consequences. First, controller behavior becomes inherently stepwise: dose decisions are tied to the most recent cuff value and may be outdated by the time actuation occurs. Second, measurement failures (movement, shivering, cuff malposition, failed inflation cycles) can disproportionately affect safety because there is no parallel beat-to-beat backup stream [18,19]. Even when average control metrics appear acceptable, intermittent systems can under-detect short but clinically relevant hypotensive episodes.

For these reasons, NIBP-based automation is best viewed as a conservative closed-loop implementation suited to lower-acuity settings, or as a fallback channel in hybrid designs. In such contexts, robust safeguards are essential: missed-cycle alarms, conservative dose-step limits between cycles, and explicit clinician notification when two consecutive measurements are unavailable.

### 4.2. Continuous Noninvasive Arterial Pressure (Cnap/Nexfin)

Continuous noninvasive arterial pressure platforms provide beat-to-beat data without arterial cannulation and therefore represent an important translational bridge between simple intermittent monitoring and invasive control environments [16,17,26]. In published systems, CNAP and Nexfin/ClearSight enabled faster decision cycles and facilitated dual-vasopressor logic that would be difficult to sustain using intermittent cuff measurements alone [21,22,29,31].

However, continuity does not guarantee stability. Finger-cuff technologies remain susceptible to motion artifacts, vasoconstriction-related signal attenuation, peripheral perfusion variability, and periodic recalibration interruptions [16,17,26]. These failure modes are especially relevant during vasoactive titration, when peripheral vascular tone can change dynamically and bias pressure reconstruction. In practice, this creates a paradox: the modality offers richer temporal information, yet may intermittently degrade exactly when the controller is most reliant on accurate beat-to-beat input.

Comparative observations from DIVA/ADIVA-era studies illustrate this point: advanced control logic operating on noisier or unstable input may amplify variability into unnecessary dose oscillation [16,17,26]. Therefore, continuous noninvasive monitoring should be coupled with explicit quality gating, artifact rejection, and fallback policies rather than being treated as a drop-in surrogate for arterial waveform reliability.

### 4.3. Invasive Arterial Pressure Monitoring

Invasive arterial monitoring remains the most robust input modality for high-precision closed-loop vasopressor titration, particularly in major surgery and critical care cohorts where rapid hemodynamic transitions are expected [20,21,23,25,27,28,29]. Beat-to-beat waveform availability with minimal latency supports tighter control bandwidth, more stable PID behavior, and more reliable time-in-target performance in reported clinical implementations [20,23,24,26,32].

Importantly, “invasive” does not mean “error-free.” Waveform damping, resonance, transducer leveling errors, and flush-related disturbances can still introduce clinically meaningful artifacts if not actively managed. Nonetheless, these issues are typically detectable through waveform-quality checks and standardized line management, making them operationally more controllable than unpredictable dropout in some noninvasive modalities [23,27].

From a deployment perspective, invasive monitoring is not universally indicated and therefore cannot be the default pathway for all patients. Instead, it should be considered the preferred input when therapeutic precision requirements are high, hemodynamics are highly labile, or pharmacodynamic consequences of delayed correction are substantial. A practical selection framework is to match sensor modality to both clinical risk and controller complexity: more complex or highly dynamic control strategies require more reliable and lower-latency inputs [9,10].

These modality-specific constraints directly determine downstream requirements for filtering, quality gating, and latency-aware decision support, which are addressed in the next section.

## 5. Signal Quality, Preprocessing, and Data Integrity

Selecting the right sensor modality, as described in Section 4, is only the first step. Even a high-quality sensor produces raw pressure values that are not yet equivalent to control-grade data [9,10,14]. Between acquisition and dosing, signals undergo filtering, plausibility checks, temporal alignment, and confidence assessment. The quality of this preprocessing layer can determine whether a controller behaves as a stabilizing or destabilizing element, especially when operating near physiological thresholds [9,10].

A useful way to structure this domain is by three data-quality threats: artifact, drift, and delay. Artifacts include transient nonphysiological excursions (e.g., movement- or line-related spikes) that can trigger inappropriate dose commands if not rejected. Drift represents gradual bias in pressure estimation (e.g., calibration shifts or vascular tone-dependent distortion in noninvasive systems), which may produce persistent under- or over-treatment despite apparently smooth control curves. Delay includes sampling intervals, processing time, transport latency, and actuator lag; accumulated delay narrows the safe control bandwidth and can induce oscillatory behavior [16,17,18,23,25,26,27,28,29,30].

Minimum preprocessing requirements should therefore include: (1) range and slope plausibility checks; (2) outlier rejection with short persistence windows; (3) signal-quality indices visible to both algorithm and clinician; (4) timestamp integrity controls; and (5) explicit missing-data policies. Missing-data handling deserves particular emphasis: “last value held” may be acceptable for very short gaps but becomes hazardous when applied across prolonged dropout, where stale values can drive false confidence in loop stability.

Data integrity should also be treated as a reporting endpoint, not only an internal engineering detail. Studies should report sampling frequency, effective controller update interval, preprocessing logic, rate of invalidated samples, dropout burden, and latency from measurement to command execution. Without these parameters, comparative interpretation of performance metrics (e.g., MDAPE, time-in-target, hypotension burden) remains incomplete and potentially misleading [9,10]. In short, controller outcomes are inseparable from the quality-governance layer that precedes decision logic.

In practical terms, the preprocessing layer defines the effective operating envelope of the controller. Understanding its requirements is inseparable from selecting the right control architecture, the subject of the following section.

## 6. Control Architectures and Decision Logic

Control architecture determines how measured pressure deviations are converted into vasopressor commands, but real-world performance emerges from the interaction between architecture, sensor behavior, and pharmacodynamic delay [9,10]. Published systems span on–off logic, proportional control, PID formulations, rule-based multi-drug strategies, and multi-agent frameworks, each reflecting different trade-offs between simplicity, interpretability, responsiveness, and implementation burden [16,17,18,19,20,21,22,23,24,25,26,27,28,29,30].

At one end, simple controllers (on–off or fixed proportional rules) are transparent and easy to implement, with fewer tunable parameters and lower risk of hidden instability. Their limitations are reduced adaptability and potential chattering around targets when input variability is high. At the other end, PID and multi-agent systems can achieve tighter control under reliable input conditions but require careful tuning, anti-windup safeguards, saturation handling, and clinically coherent fail-safe rules [20,23,24,26,27,28,30,33].

A key translational insight is that algorithmic complexity should be matched to information quality and clinical context, not pursued as an independent objective. In lower-risk scenarios with sparse or moderately noisy data, conservative and interpretable logic may outperform more aggressive adaptive schemes by reducing overreaction to uncertainty. In higher-risk settings with invasive beat-to-beat input, more advanced controllers become both feasible and clinically justifiable because the signal supports higher control bandwidth and finer dose modulation [10,11,20,23,32].

Another practical distinction is between single-agent and multi-agent control. Single-agent norepinephrine systems simplify attribution of effect and reduce interaction complexity. Multi-agent platforms may better mirror clinician reasoning in selected environments but introduce additional interaction terms, tuning dependencies, and supervisory demands that can hinder scalability outside specialized teams [27]. For comprehensive review purposes, controller performance should therefore be interpreted within a triad: input fidelity, control logic, and implementation ecosystem.

### 6.1. Algorithmic Families Reported in the Literature

**Figure 2:**
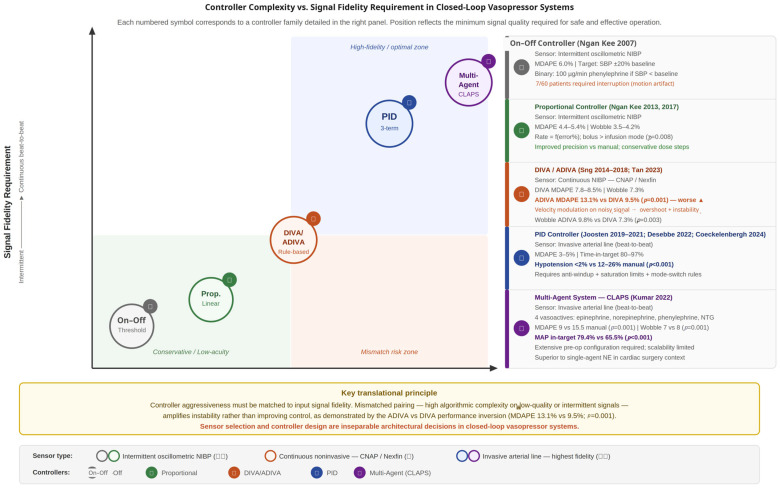
Controller complexity vs. signal fidelity requirement in closed-loop vasopressor systems. Each symbol corresponds to a controller family positioned according to the minimum signal quality required for safe operation. Circle area reflects the breadth of clinical evidence. The mismatch risk zone (lower right) represents combinations of high controller complexity with low signal fidelity; the ADIVA experience is annotated as a key example of this failure pattern.

Closed-loop systems employed diverse algorithmic architectures, which we categorize into five principal families based on control logic: (1) on–off controllers, (2) proportional controllers, (3) rule-based dual-vasopressor systems, (4) proportional–integral–derivative (PID) controllers and (5) multi-agent systems. Representative controller families and clinical implementations are summarized in Supplementary Table S2, and their positioning relative to signal fidelity requirements is illustrated in Figure 2.

On–Off Controllers: The earliest implementation [18] used simple threshold-based binary logic: phenylephrine infusion (100 μg/min) was initiated when systolic blood pressure (SBP) fell below baseline and stopped when SBP exceeded baseline. This approach achieved 94.6% of measurements within ±20% of target but exhibited characteristic oscillatory behavior inherent to on–off control, with MDAPE of 6.0%. The system required interruption in 7/60 patients due to monitoring failures, predominantly motion artifacts from shivering compromising intermittent noninvasive measurements.

Proportional Controllers: Subsequent work [16,18] introduced proportional control where infusion rate scaled linearly with pressure deviation: I = (10 − error%) × 3 mL/h, bounded between 0 and 60 mL/h. This strategy demonstrated improved precision (MDAPE 4.38–5.39%) and reduced oscillation (wobble 3.5–4.2%) compared with manual control. A key finding was that intermittent bolus delivery (calculating 1 min dose and administering as rapid bolus) achieved superior performance versus continuous infusion (MDAPE 4.38% vs. 5.39%, *p* = 0.008), likely by synchronizing drug delivery with measurement updates and preventing infusion-during-measurement errors that inflated effective dose [17].

Rule-Based Dual-Vasopressor Systems (DIVA/ADIVA): The DIVA platform [21,29,31] employed conditional logic mimicking clinical decision-making: phenylephrine for isolated hypotension (SBP < 90% baseline AND HR ≥ 60 bpm); ephedrine for hypotension with bradycardia (SBP < 90% baseline AND HR < 60 bpm). Performance varied with monitoring technology: DIVA with CNAP yielded MDAPE 8.5%, while DIVA with Nexfin achieved MDAPE 7.8% [16,17]. The advanced iteration ADIVA added velocity-modulation logic, adjusting bolus speed based on SBP trend (rapid bolus for declining pressure, slow infusion for rising pressure). Contrary to design intent, ADIVA demonstrated inferior performance (MDAPE 13.1% vs. 9.5% for DIVA, *p* = 0.001) with increased wobble (9.8% vs. 7.3%, *p* = 0.003), suggesting that aggressive trend-based acceleration introduced overshoot and instability [22].

PID Controllers: Studies in major surgery and critical care universally employed PID control for norepinephrine titration [23,24,25,26,28,29]. The PID algorithm computed infusion rate adjustments based on three terms: proportional (current error magnitude), integral (cumulative error over time), and derivative (rate of pressure change). This architecture, augmented with safety rules limiting rate-of-change and absolute dose bounds, demonstrated excellent performance: MDAPE 3–5% [20,23,30], time-in-target 80–97% [20,26], and hypotension < 2% in closed-loop versus 12–26% in manual control [24,28]. The superior performance of PID systems likely reflects algorithmic sophistication and the use of invasive arterial monitoring, which eliminates finger-cuff-related signal artifacts [23,27].

Multi-Agent Systems: The CLAPS platform [23], which simultaneously titrated epinephrine, norepinephrine, phenylephrine, and nitroglycerin according to MAP and heart-rate targets, demonstrated superior performance during cardiac surgery. It maintained MAP within ±20% of target for 79.4% vs. 65.5% of case time (*p* < 0.001) and achieved significantly better precision, with lower MDAPE (9 vs. 15.5; *p* = 0.001) and wobble (7 vs. 8; *p* = 0.001) compared with manual control. However, despite these improvements, the system required extensive preoperative configuration and continuous intraoperative oversight, substantially limiting its scalability and real-world clinical applicability.

### 6.2. Controller Behavior Under Noisy or Delayed Inputs

Controller behavior under uncertainty is central to patient safety. Two phenomena dominate: noise amplification and delay-induced instability. Noise amplification is particularly relevant for derivative-sensitive or trend-reactive logic, where beat-to-beat variability can be misclassified as physiological deterioration, leading to overshoot and oscillation. Delay-induced instability occurs when dosing decisions are based on outdated state information, causing the loop to “chase” prior errors rather than current physiology [9,10].

Different controller families fail differently under these conditions. On–off controllers tend to produce threshold chattering when measurements fluctuate around setpoints [18]. Proportional controllers may overcorrect if gain is high relative to signal noise. PID controllers can exhibit integral windup during actuator limits or dropout intervals unless anti-windup constraints are implemented [23,25,27,28]. Trend-accelerated strategies may be especially vulnerable when velocity estimates are derived from noisy noninvasive inputs, as illustrated by performance degradation in advanced dual-vasopressor implementations [22].

Delay compounds these effects by reducing phase margin. In practical terms, delays from intermittent sampling, filtering, middleware transport, and pump actuation can accumulate to a point where otherwise stable gains become oscillatory. This is why control tuning cannot be separated from the measured end-to-end latency budget. A controller validated at one delay profile may become unsafe when deployed on a different monitor-pump stack with longer command paths [10,11,16,17,28].

Mitigation should be explicit and layered: gain scheduling by signal confidence, deadbands/hysteresis near target, minimum command dwell times, anti-windup, command-rate limits, and forced de-escalation to conservative modes when quality or latency thresholds are breached. Importantly, these are not optional refinements; they are core safety mechanisms that convert theoretical control capability into clinically reliable behavior.

Controller stability, in other words, is necessary but not sufficient: even a well-tuned algorithm fails if the integration layer cannot reliably execute its commands, as discussed in the following section.

## 7. System Integration, Actuation, and Human-in-the-Loop Supervision

Closed-loop vasopressor performance is fundamentally a system-integration problem. Even with high-fidelity sensing and well-tuned algorithms, failures in monitor–controller–pump communication can degrade or negate therapeutic benefit [23,27]. Integration quality depends on deterministic data flow, robust message acknowledgment, unit consistency, timestamp synchronization, and fail-safe behavior when any component becomes unavailable.

Actuation design requires similar scrutiny. Bolus versus infusion semantics, minimum update intervals, and dose saturation limits all influence loop stability and safety. For example, frequent, small updates may improve precision but can increase communication load and sensitivity to transient noise; less frequent, larger adjustments may reduce chatter, but risk delayed correction during rapid instability. These trade-offs should be made explicit and justified relative to clinical context and sensor latency characteristics [16,18,23,24,33].

Human supervision remains essential across current platforms. “Human-in-the-loop” should not be interpreted as a binary state (manual versus autonomous), but as a graded architecture ranging from advisory systems to semi-closed-loop confirmation models to high-autonomy controllers with emergency override [12,30]. Semi-closed-loop designs reduce automation risk by requiring clinician confirmation but may also reintroduce reaction delays and workload burden. High-autonomy designs can improve responsiveness but require strong transparency, alarm prioritization, and trust calibration to avoid out-of-the-loop performance [12,13]. A complementary framing is the concept of the “exportable expert”: by encoding optimized hemodynamic decision protocols into automated platforms, closed-loop systems enable practitioners with limited experience to achieve management quality approaching that of expert anesthesiologists, thereby reducing outcome variability across providers and institutions. This standardization benefit operates independently of whether a full closed-loop or decision-support architecture is used, and represents a compelling argument for the clinical translation of these systems beyond centers of excellence.

Accordingly, clinical deployment should include predefined supervision protocols: who monitors loop status, what triggers manual takeover, how overrides are documented, and how teams are trained for failure scenarios. Reporting should include override frequency, reasons for takeover, alarm-response time, and integration incidents, since these variables directly determine real-world safety and scalability [19,23,28].

This system’s perspective motivates the reliability analysis below, where recurrent failure pathways are organized by domain and linked to practical mitigation strategies.

## 8. Reliability Vulnerabilities and Failure Modes

Although closed-loop vasopressor systems can show favorable average control performance, bedside safety depends on how the system behaves under uncertainty, stress, and partial failure. In this context, reliability means more than maintaining target pressure during ideal operation; it means preserving clinically coherent behavior when signals deteriorate, delays accumulate, or communication pathways intermittently fail. A reliability-focused assessment therefore considers fault detectability, degradation behavior, recovery dynamics, and safety of manual takeover pathways, not only nominal endpoint metrics [10,11,23,28].

For practical analysis, vulnerabilities can be organized into three interacting domains: sensor-related failures, algorithm-related instabilities, and system-integration errors. This structure is clinically useful because similar hemodynamic outcomes (e.g., prolonged hypotension, overshoot, unstable dosing) may arise from different root causes, each requiring distinct mitigation strategies. For example, transient pressure dropout, unstable gain behavior under noisy input, and delayed pump-command execution can all converge on the same adverse pressure trajectory [10,11,23,28].

### 8.1. Sensor-Related Failures

Sensor reliability remains the dominant determinant of loop stability. Across reported implementations, finger-cuff platforms were frequently represented among technical interruptions, particularly under motion, shivering, edema, vasoconstriction, or reduced peripheral perfusion [16,17,26]. CNAP-type systems may experience transient signal loss or recalibration interruptions, while Nexfin/ClearSight platforms generally provide improved continuity but remain vulnerable to similar physiologic and motion-sensitive interferences [16,17,26].

Intermittent oscillometric monitoring presents a different reliability profile. Rather than abrupt dropout, it introduces predictable measurement gaps (commonly around one minute in obstetric workflows), creating dead-time in which control actions rely on potentially stale pressure information [18,19,30]. This can limit responsiveness during rapid hemodynamic transitions and may permit short but clinically relevant hypotensive episodes between cuff cycles.

Invasive arterial monitoring demonstrated the most stable input profile in published perioperative studies, particularly in higher-acuity settings where tighter MAP control was pursued [20,24,26,28,32,33]. While invasive waveforms can still be affected by damping, leveling errors, or line artifacts, these issues are often operationally detectable and manageable through standardized waveform-quality checks. Overall, sensor selection should be matched to expected hemodynamic volatility and to the intensity of the intended control strategy [9,10].

### 8.2. Algorithm-Related Instabilities

Controller behavior under non-ideal input is a central reliability concern. Simple threshold-based logic can produce chattering near setpoints, while proportional and PID-based systems may overshoot when gains are aggressive relative to signal noise or delay [18,23,25,27,28,29]. Integrator windup and oscillatory behavior become more likely when actuator limits are reached or when noisy measurements are interpreted as sustained error.

Importantly, increased algorithmic complexity does not automatically translate into improved clinical robustness. In obstetric dual-vasopressor implementations, the ADIVA platform showed higher MDAPE and wobble than earlier DIVA configurations, illustrating that trend-based “intelligence” may amplify measurement uncertainty when signal fidelity is limited [22]. To clarify the distinction, DIVA (Double Intravenous vasopressor Automated system) used a rule-based logic selecting between phenylephrine and ephedrine based on MAP and HR thresholds, operating on continuous noninvasive (Nexfin/CNAP) input. ADIVA (Advanced DIVA) added a velocity modulation layer that accelerated or decelerated bolus delivery based on the rate-of-change of SBP trend. In theory, trend-reactive acceleration should improve recovery speed; in practice, when applied to noisy noninvasive signals, this added layer introduced overshoot and oscillation, ultimately worsening control metrics compared with the simpler parent architecture. This illustrates a counterintuitive but important principle: in closed-loop systems, additional algorithmic intelligence can degrade performance when the input signal does not reliably support the assumptions underlying that intelligence. This supports a practical translational principle: controller complexity should be matched to input quality and latency characteristics, with conservative fallback behavior when confidence degrades [10,11].

From a safety perspective, algorithm performance should therefore be interpreted together with preprocessing design (artifact rejection, plausibility filters, quality gating) and with explicit command constraints (step limits, dwell times, anti-windup, de-escalation rules). These safeguards are not secondary refinements; they are core determinants of whether theoretical control capability is converted into reliable bedside behavior.

### 8.3. System-Integration Errors

Whereas sensor and algorithm failures tend to manifest in control metrics, integration errors are more insidious: they arise at the interfaces between monitor, controller software, middleware, and infusion pump, yet may produce identical adverse outcomes through an entirely different mechanism. Reported events include communication failures linked to third-party drivers or serial/protocol mismatches, resulting in delayed or failed dose updates [23,27]. Even when direct patient harm is not documented, such events highlight a key systems property: closed-loop reliability is limited by the weakest link in the monitor–controller–actuator chain.

Integration reliability depends on deterministic data flow, timestamp integrity, command acknowledgment, and fail-safe behavior during communication loss. Without these safeguards, minor interface disruptions can cause clinically significant treatment delays or unsafe persistence of outdated commands. This risk is especially relevant in heterogeneous device ecosystems where components are sourced from different vendors and validated separately.

Supervisory design is tightly coupled with integration reliability. Semi-closed-loop workflows requiring clinician confirmation can reduce certain automation risks but may also introduce response delays and workload dependence during instability [32]. Accordingly, studies should report override criteria, takeover timing, alarm hierarchy, and mode-transition behavior with the same rigor used for efficacy endpoints.

### 8.4. Cross-Domain Interactions and Mitigation Priorities

In real-world operation, failures are often cross-domain rather than isolated. Sensor degradation can provoke algorithmic oscillation; unstable command patterns can expose integration fragility; and delayed supervisory response can amplify either process. Reliability evaluation should therefore prioritize interaction pathways, not only single-component defects.

A layered mitigation strategy is recommended: real-time signal-quality gating, latency-aware tuning, anti-windup and command-rate limits, communication watchdog/heartbeat checks, explicit fallback modes, and predefined manual takeover triggers [9,10,23,27]. Together, these measures enable graceful degradation, maintaining safe behavior when ideal closed-loop control is temporarily unattainable.

Overall, closed-loop vasopressor safety should be treated as an emergent property of the full monitoring–decision–actuation ecosystem. This three-domain framework provides a practical basis for the mitigation matrix in Table 2 and for the validation-oriented interpretation developed in the next section.

## 9. Clinical Validation Landscape and Benchmarking

The reliability framework developed in Section 8 provides a lens through which to read the clinical validation literature. Validation of closed-loop vasopressor systems has expanded across obstetric anesthesia, major noncardiac surgery, postoperative high-risk pathways, and limited critical care contexts, with meaningful heterogeneity in monitoring inputs, controller logic, supervision models, and endpoint definitions.

From a translational perspective, available studies indicate that closed-loop platforms have been associated with improved hemodynamic stability metrics in multiple environments, although external validity remains uneven across settings and workflows [17,18,20,24,26,27,28,29,32,34,35]. Studies conducted in tightly controlled workflows with higher-fidelity monitoring tend to report more consistent control behavior, whereas broader real-world contexts show greater variability related to signal reliability, clinician interaction, and integration dependencies [10,11,23,28]. Accordingly, clinical validation is best interpreted as context-dependent system performance rather than as a universal property of closed-loop control as a generic category.

### 9.1. Validation Patterns by Clinical Setting

In obstetric anesthesia, validation pathways are comparatively mature, supported by repeated implementations of phenylephrine-centered and dual-vasopressor strategies using intermittent and continuous noninvasive monitoring [16,17,18,21,22,29]. These studies are particularly informative for feasibility, workflow integration, and target-tracking behavior under controlled but physiologically dynamic conditions. However, extrapolation to broader perioperative populations should be cautious because obstetric physiology, target definitions, and intervention timing differ from major surgical and ICU contexts.

In major surgery and postoperative high-risk cohorts, validations more commonly use invasive arterial input and tighter MAP control paradigms, providing important evidence for precision-oriented automation in hemodynamically labile patients [20,23,24,26,28,32,33]. These environments better stress-test controller robustness to rapid perturbations, but also increase dependence on reliable integration and alarm/override workflows. As a result, technical readiness in these settings should be judged by both endpoint performance and operational resilience under non-ideal conditions [23,27].

### 9.2. Benchmarking Metrics and Comparability

A central challenge in the current landscape is non-uniform endpoint selection. Commonly reported metrics include time-in-target pressure, hypotension burden, variability/overshoot behavior, and error-based indices such as MDPE/MDAPE and wobble/divergence [10,11,16,17,18,20,21,22,23,24,26,28,29,31,32,33]. While individually useful, these measures are not interchangeable. For example, favorable time-in-target can coexist with undesirable oscillation, and low median error may mask intermittent but clinically important excursions.

Accordingly, benchmark interpretation should move from single-metric judgment to multidomain profiles combining precision, stability, safety, and reliability reporting. At minimum, studies should pair physiological endpoints (e.g., hypotension burden) with technical reliability indicators (e.g., signal dropout, communication interruptions, override frequency, and recovery behavior), so that apparent efficacy can be interpreted considering system robustness [10,11,23,28].

### 9.3. Interpreting Outcomes Through the Reliability Lens

Section 8 highlights that similar hemodynamic outcomes can emerge from different failure pathways. This principle is directly relevant when reading validation studies: superior endpoint performance in one platform may reflect stronger sensing and integration rather than inherently better decision logic, whereas underperformance may result from input instability rather than flawed controller design [10,11]. Therefore, comparative interpretation should explicitly account for monitoring modality, preprocessing strategy, latency budget, and supervisory architecture before attributing benefit or harm to algorithm class alone.

This reliability-aware interpretation is especially important when advanced controllers are deployed on noisier or interruption-prone input streams. In such situations, algorithmic sophistication may not translate into clinical robustness unless matched by high data integrity and conservative guardrails [9,10,22]. A practical implication is that validation reports should document not only “what the controller did,” but “what information quality and system conditions it depended on to do it safely.”

### 9.4. Gaps and Translational Priorities

Despite encouraging validation signals, several translational gaps remain. First, cross-setting generalizability is limited by heterogeneous targets, inconsistent technical disclosures, and variable reporting of failure events. Second, reliability outcomes are often underreported relative to efficacy endpoints, reducing confidence in deployment readiness. Third, human-factor variables (override burden, alarm handling, takeover latency) are frequently described qualitatively rather than benchmarked quantitatively, despite their direct impact on safety and scalability [12,13,32].

To address these gaps, future validation should follow standardized reporting frameworks that integrate sensor quality, control behavior, integration integrity, and supervision performance into unified outcome models (see Box 1 and Table 2). This approach would allow systematic evidence mapping to evolve from descriptive comparison toward reproducible, implementation-oriented benchmarking across institutions and device ecosystems. These validation patterns and persisting evidence gaps directly motivate the reporting framework proposed in the next section, aimed at improving comparability, reproducibility, and implementation safety across closed-loop vasopressor studies.

Box 1 **Minimum reporting checklist for closed-loop vasopressor studies.**

(1) Clinical context and intended use. Report care setting (OR/ICU/obstetrics), procedure type, population risk profile, target MAP strategy, and vasopressor(s) used; clarify whether the system is decision-support or autonomous titration with clinician supervision.
(2) Monitoring input (sensor modality and configuration). Specify arterial line vs. continuous/intermittent noninvasive BP, device make/model, sampling and update rates, calibration/recalibration behavior, and known sensor limitations relevant to vasoconstriction, motion, or perfusion.
(3) Signal quality and preprocessing. Describe filtering, artifact detection/rejection, plausibility checks, signal-quality indices/flags, handling of missing data, and total measurement-to-command latency.
(4) Control-algorithm specification. Declare control family (rule-based, PID, adaptive, etc.), target tracking method, tuning approach, anti-windup or damping logic, dose-step limits, lockout periods, and escalation/de-escalation rules.
(5) Actuation and device integration. Identify infusion pump platform, command transmission method, update interval, closed-loop cycle time, and communication verification/handshake procedures.
(6) Safety supervision and override logic. Provide hard/soft safety bounds, alarm hierarchy, override criteria, fallback-mode behavior, and conditions that trigger transition to manual control.
(7) Human factors and workflow integration. State clinician roles, training protocol, interface/display used, documented workload impact, and override frequency/reasons.
(8) Performance metrics (technical + physiological). Report time-in-target MAP, hypotension burden, overshoot/variability, MDPE/MDAPE/wobble/divergence (or equivalent), and command-stability metrics.
(9) Reliability and failure reporting. Use a predefined failure taxonomy (sensor failure, algorithm instability, integration error), with event frequency, duration, detection pathway, mitigation, and recovery time.
(10) Clinical outcomes and harm. Report predefined clinical endpoints and adverse events, including vasoactive drug burden, rescue interventions, and complications potentially related to automation.
(11) Study design and data transparency. Describe study design/comparator, allocation and blinding methods when applicable, protocol deviations, missing-data handling, and statistical analysis plan.
(12) Reproducibility essentials. Provide software/firmware version, parameter set used in the study, and sufficient methodological detail to reproduce controller behavior across sites/devices.

### 9.5. Limitations and Comparative Context

Closed-loop vasopressor systems offer meaningful advantages over conventional manual titration in specific contexts, but these benefits are neither universal nor unconditional. Understanding when automated systems outperform manual approaches, and when they do not, is essential for appropriate clinical deployment and for interpreting the existing validation literature [10,11,13,14].

Compared with fixed-rate vasopressor infusion protocols (a common alternative in resource-limited or obstetric settings), closed-loop systems provide dynamic dose adjustment that better tracks individual hemodynamic variability. However, fixed protocols have documented safety in low-acuity populations and are substantially easier to implement without specialized hardware or training [18,19,30]. Compared with nurse-driven titration in ICU or postoperative settings, closed-loop platforms reduce reaction delay and attenuate operator workload during sustained instability, but introduce new failure modes related to sensor reliability and device integration that are absent in purely human-driven workflows [20,21,28]. Neither approach is inherently superior; the appropriate choice depends on clinical risk, monitoring infrastructure, available expertise, and the degree of hemodynamic lability anticipated.

Several deployment barriers further constrain the generalizability of existing evidence. First, most validated platforms require proprietary hardware interfaces or custom software integration that is not readily available outside research environments, limiting scalability to routine clinical settings [23,27]. Second, regulatory pathways for closed-loop vasopressor devices remain incompletely defined in most jurisdictions, creating uncertainty about approval requirements and post-market surveillance obligations. As noted by Coeckelenbergh and colleagues, the chief difficulty lies in ensuring patient safety with agents that have a very narrow therapeutic margin, where consequences of overdose are severe, and this challenge does not make closed-loop control of vasoactive agents impossible, but the combined design, safety, and regulatory burden has historically outweighed the commercial incentive for industry investment. This may be changing as cost-containment pressures increasingly drive payers and regulators to seek consistent, protocol-driven outcomes. Third, training requirements for safe operation, including failure recognition, override protocols, and alarm management, are rarely quantified in validation studies, yet likely represent a significant implementation burden in high-turnover clinical teams [12,13,32]. The broader challenge of deploying AI-assisted clinical tools in anesthesiology, including the need for multi-domain evaluation frameworks encompassing performance, usability, human factors, and governance, applies directly to closed-loop vasopressor systems, which must satisfy clinical, regulatory, and operational criteria before widespread adoption [36]. Fourth, cost-effectiveness data are essentially absent, making health-system adoption decisions difficult to justify on an evidence basis beyond clinical performance metrics alone.

The narrative and architecture-centered synthesis used in this review is intentionally broad, prioritizing mechanistic generalizability over pooled effect estimation. This approach is appropriate given the heterogeneity of platforms, monitoring modalities, and clinical contexts represented in the literature, but it means that effect size quantification and formal risk-of-bias assessment, as would be performed in a systematic review or meta-analysis, are outside the scope of this work. Future reviews with more homogeneous platform-specific datasets may be able to address those questions with greater precision [9,10].

## 10. Reporting and Validation Recommendations

To improve comparability, reproducibility, and safety inference, reporting should progress from “algorithm description” to a full system-of-systems disclosure. At minimum, studies should provide complete information on input modality, preprocessing, decision logic, actuation pathways, and supervisory safeguards, consistent with the checklist in Box 1 [10,11,16,20,21,23,24,26,27,28,29,32,33]. Without these elements, favorable performance metrics cannot be reliably generalized across institutions or device stacks. This reporting philosophy aligns with the multi-criteria implementation framework recently proposed for AI tools in anesthesiology, which identifies performance, usability, human factors, and responsible governance as co-equal prerequisites for clinical deployment; the four-domain framework above operationalizes these principles specifically for closed-loop vasopressor systems.

## 11. Conclusions

Prior to this review, no unified framework linked sensor modality, signal preprocessing, controller architecture, and system integration as co-determinants of closed-loop vasopressor performance and safety. Individual studies reported efficacy metrics but rarely characterized the technical conditions under which those metrics were achieved, making cross-platform comparison and deployment generalizability difficult. The heterogeneity of monitoring inputs, control algorithms, and supervisory models had not been systematically organized into a translational failure-mode taxonomy. 

This review addressed those gaps by synthesizing the major closed-loop vasopressor architectures reported in the literature through an end-to-end information pipeline lens, from monitoring input through signal conditioning, decision logic, actuation, and supervisory safety layers. We demonstrated that sensor modality is not a background variable but a primary determinant of control bandwidth, reliability, and failure behavior: intermittent oscillometric devices introduce predictable dead-time that limits responsiveness; continuous noninvasive platforms improve temporal resolution but require explicit quality gating; invasive arterial monitoring provides the highest control fidelity but imposes access requirements that restrict universal applicability. We further showed that increased algorithmic complexity does not automatically confer clinical benefit, as the ADIVA experience illustrates that trend-reactive intelligence can amplify measurement noise rather than improve stability when input signal quality is limited. Finally, we organized recurrent reliability vulnerabilities into a three-domain taxonomy (sensor failures, algorithm instabilities, integration errors) and translated these into a minimum reporting checklist (Box 1) and a mitigation matrix (Table 2) to support standardized future validation. 

The primary implication is that closed-loop vasopressor safety should be treated as an emergent property of the full monitoring–decision–actuation ecosystem, not as an attribute of the algorithm alone. Achieving consistent clinical performance requires deliberate matching of sensor modality to controller complexity, explicit data-quality governance prior to command generation, and layered fail-safe architecture across all integration interfaces. A secondary implication is that the current validation landscape is insufficient for deployment generalizability: efficacy signals are encouraging, but reliability outcomes, failure event documentation, and human-factor benchmarks remain systematically underreported. Future development should therefore prioritize three directions. 

First, standardized outcome frameworks that pair physiological endpoints with technical reliability indicators (signal dropout rates, integration incidents, override burden) are needed to allow reproducible cross-platform benchmarking. Second, validation pathways should follow staged progression from bench simulation to controlled feasibility testing to real-world deployment monitoring, with predefined failure taxonomies applied consistently at each stage. Third, as artificial intelligence and adaptive learning architectures enter this domain, the principles established here (input signal reliability, transparency of control logic, graceful degradation under uncertainty) will remain foundational requirements regardless of algorithmic sophistication, and should be evaluated using multi-domain frameworks that extend beyond single performance metrics to encompass usability, safety, and governance [36]. 

Closed-loop vasopressor systems have the potential to become a cornerstone of precision perioperative hemodynamic management; realizing that potential depends on building the sensor-to-actuation evidence base with the same rigor currently applied to efficacy endpoints.

## Figures and Tables

**Table 1:** Monitoring modalities for closed-loop vasopressor control. Sampling/update characteristics, common artifacts or dropout causes, data-quality vulnerabilities, implications for automated control, and recommended safeguards across six monitoring strategies.

Monitoring Modality	Typical Sampling/Update Characteristics	Common Artifacts/Dropout Causes	Data-Quality Vulnerabilities	Implications for Automated Control	Recommended Safeguards for Closed-Loop Use
Intermittent oscillometric NIBP	Discrete cuff cycles (e.g., every 1–5 min); no beat-to-beat continuity between measurements	Cuff motion, malposition, prolonged inflation intervals, failed cuff cycles	Temporal sparsity; stale values between cycles; missed short hypotensive episodes	Delayed correction and reduced responsiveness; vulnerable to under-recognition of rapid hemodynamic changes	Latency-aware conservative tuning; hold logic between cycles; mandatory trend confirmation before large dose changes; fail-safe alarm for missed cycles
Continuous noninvasive arterial pressure (CNAP; volume clamp/finger cuff)	Near-continuous waveform with periodic recalibration/physical interruptions	Motion artifact, peripheral vasoconstriction, poor finger perfusion, cuff displacement, recalibration dropouts	Transient signal loss and drift; reduced reliability in low-perfusion states	May support tighter control than intermittent NIBP, but performance can degrade when signal quality deteriorates	Real-time signal-quality indices; artifact rejection; fallback mode during recalibration/dropout; periodic reference checks
Nexfin/ClearSight-like finger arterial pressure systems	Continuous pressure estimation with device-specific filtering and calibration behavior	Peripheral vasoconstriction, movement, finger size/position mismatch, calibration drift	Bias/drift risk under vasoactive states; intermittent instability during calibration cycles	Can enable closed-loop titration in selected settings but requires robust quality gating to prevent erroneous dosing	Quality-threshold gating; automatic suppression of aggressive commands under low confidence; predefined manual override triggers
Invasive arterial line (A-line)	Beat-to-beat high-frequency waveform (typically highest temporal resolution)	Line damping/resonance, air bubbles, catheter kinking/flush events, transducer leveling/zeroing errors	Waveform distortion if setup is suboptimal; calibration/leveling errors can create systematic bias	Most suitable for high-precision closed-loop control due to continuity and fidelity when line quality is maintained	Dynamic waveform-quality checks (e.g., damping/fast-flush assessment); transducer setup protocol; plausibility filters and alarmed quality degradation
Hybrid input strategy (primary continuous + backup intermittent NIBP)	Continuous stream with intermittent cross-checks or fallback measurements	Discordance between devices, asynchronous timestamps, backup cycle failures	Cross-modality inconsistency and timing mismatch	Improves resilience if switching logic is robust; may reduce unsafe operation during primary signal loss	Explicit input-priority hierarchy; timestamp synchronization; discordance thresholds triggering supervisory review/manual mode
Postoperative/ICU monitor integrations (multi-device data stream)	Continuous input potentially subject to network and middleware delays	Packet loss, timestamp desynchronization, interface handshake errors	Latency accumulation and stale command generation despite apparently continuous data	Controller may react to outdated state if transport delays are not accounted for	End-to-end latency monitor; watchdog/heartbeat checks; command acknowledgment; automatic transition to safe mode when delay limits are exceeded

Abbreviations: A-line, arterial line; CNAP, continuous noninvasive arterial pressure; NIBP, noninvasive blood pressure. Practical recommendation: studies should explicitly report sampling frequency, effective update interval, recalibration behavior, latency from measurement to actuation, and signal-quality handling rules.

**Table 2:** Failure modes and mitigations: summary by domain (see Supplementary Table S1 for full detail). Condensed 3-row matrix by failure domain (sensor-related, algorithm-related, integration-related) with representative failure types, key mechanisms, main clinical risks, and core mitigations.

Failure Domain	Primary Failure Types	Key Mechanisms	Main Clinical Risks	Core Mitigations
**Sensor-related**	Signal dropout; implausible values; calibration drift/bias	Motion artifact; vasoconstriction; cuff failure; line disturbance	Absent or erroneous vasopressor command; prolonged hypotension; persistent MAP error	Quality index; plausibility filter; auto hold to safe mode; forced recalibration
**Algorithm-related**	Integrator windup; threshold chattering; noise amplification (ADIVA pattern)	Actuator saturation; setpoint oscillation; trend logic on noisy signal	Dose overshoot; hemodynamic oscillation; worse performance than simpler controller	Anti-windup; deadband/hysteresis; gain reduction under low fidelity; de-escalation rule
**Integration-related**	Communication failure; unit/timestamp mismatch; delayed supervisory response	Protocol mismatch; middleware timeout; alarm fatigue; out-of-the-loop failure	Missed or incorrect dose update; uncorrected hemodynamic excursion; automation over-reliance	Watchdog/heartbeat; command acknowledgment; predefined takeover triggers; alarm hierarchy

Suggested use: Report each observed failure event with frequency, duration, detectability, mitigation applied, and recovery time.

## Data Availability

No new data were created or analyzed in this study. Data sharing is not applicable to this article.

## Supplementary Materials

The following supporting information can be downloaded at: https://www.mdpi.com/article/10.3390/s26072180/s1, Table S1: Failure Modes and Mitigations in Closed-Loop Vasopressor Systems—Complete Reference Matrix; Table S2: Curated Set of Representative Clinical Validation Studies.
