# Supplementary material for: Monitoring Inputs, Control Architectures, and Failure Modes in Closed-Loop Vasopressor Systems: A Comprehensive Review
Source: Sensors (Basel). 2026 Apr 1;26(7):2180. doi: 10.3390/s26072180 (PMC13075136; doi:10.3390/s26072180)
Supplement: Supplementary file 1 [file sensors-26-02180-s001.zip › sensors-4196353-supplementary.pdf]

Supplementary Material

Monitoring Inputs, Control Architectures, and Failure Modes in Closed-Loop Vasopressor Systems

Manuscript ID: sensors-4196353 | Sensors (MDPI) | Revision 1

Supplementary Table S1. Failure Modes and Mitigations in Closed-Loop Vasopressor Systems — Complete Reference Matrix

Full 13-row matrix organised by failure domain (sensor-related, algorithm-related, integration-related). A condensed 3-row summary appears as Table 2 in the main manuscript.

Failure Domain	Representative Mode	Mechanism / Trigger	Clinical Consequence	Detection	Mitigation
**Sensor-related**	Signal dropout / transient loss	Motion artifact; cuff recalibration; vasoconstriction; disconnection	Absent vasopressor command; prolonged hypotension	Signal-quality index; timeout alarm	Auto hold → safe mode; revalidation before re-entry
	Physiologically implausible value	Artifact spike; calibration drift; line flush disturbance	Over- or under-dosing from false pressure reading	Range/slope plausibility filter	Reject outlier; hold last valid; alert clinician
	Calibration drift / systematic bias	Vascular tone changes biasing noninvasive reconstruction	Persistent MAP error; sustained under- or over-treatment	Trend deviation alert; recalibration cycle monitoring	Force recalibration; flag sustained error; reduce dose step
	Recalibration interruption (finger-cuff specific)	Device-initiated pause for Physiocal or re-zeroing cycle	Control gap during recalibration; stale MAP input	Recalibration event flag; gap duration monitor	Conservative hold during gap; validate signal before resuming aggressive dosing
	Damped waveform / flush artifact (A-line)	Underdamping/overdamping; air bubble; flush bolus	Falsely high or low MAP; erroneous dose adjustment	Dynamic response test (fast-flush assessment)	Waveform quality checks; flush artifact rejection window; transducer level protocol

Failure Domain	Representative Mode	Mechanism / Trigger	Clinical Consequence	Detection	Mitigation
**Algorithm-related**	Integrator windup	Actuator saturation or signal dropout during active integration	Dose overshoot on signal recovery; rebound hypertension	Anti-windup flag; saturation detector	Anti-windup constraint; dose rate cap; de-escalation rule
	Threshold chattering (on-off)	Measurement fluctuation around setpoint	Oscillating vasopressor delivery; haemodynamic variability	Command frequency monitor	Deadband/hysteresis near target; minimum command dwell time
	Noise amplification by trend logic (ADIVA pattern)	Velocity-modulation applied to noisy noninvasive signal	Overshoot and instability — worse performance than simpler controller	MDAPE/wobble trend monitor	Reduce gain on noisy input; prefer conservative logic when fidelity is low
	Gain instability under delay	Accumulated latency from sampling + filtering + transport + actuation	Oscillatory behaviour; controller "chasing" prior errors	End-to-end latency monitor	Latency-aware tuning; separate gain profiles per sensor modality
**Integration-related**	Communication failure (pump interface)	Serial/protocol mismatch; middleware timeout; driver error	Delayed or failed dose update; missed therapeutic correction	Watchdog/heartbeat check; acknowledgment timeout	Fallback to last safe command; alarm; manual takeover prompt
	Unit or timestamp mismatch	Incompatible units between monitor and controller software	Dose calculation error; incorrect drug rate delivered	Unit validation on data ingestion	Hard type-checking; reject unvalidated data; alarm
	Command persistence after dropout	Last infusion rate maintained when feedback lost; no safe-mode transition	Continued or escalating vasopressor despite absent feedback	Signal-loss timeout; infusion watchdog	Automatic hold/suspend on timeout; clinician alert; rate cap during uncertainty

Failure Domain	Representative Mode	Mechanism / Trigger	Clinical Consequence	Detection	Mitigation
	Delayed supervisory response	Alarm fatigue; workload burden; out-of-the-loop failure	Uncorrected haemodynamic excursion; automation over-reliance	Override latency logging; alarm prioritization audit	Predefined takeover triggers; alarm hierarchy; team training; simulation drills

Abbreviations: ADIVA, Advanced Double Intravenous Vasopressor Automated system; A-line, arterial line; CNAP, continuous noninvasive arterial pressure; DIVA, Double Intravenous Vasopressor Automated system; MAP, mean arterial pressure; MDAPE, median absolute performance error; NIBP, noninvasive blood pressure.

Supplementary Table S2. Curated Set of Representative Clinical Validation Studies

Closed-loop vasopressor studies organised by clinical setting, sensor modality, controller family, target definition, and key reported endpoints. Intended for comprehensive-review synthesis, not pooled-effect estimation.

Study (Year)	Clinical Setting	Sensor Modality	Controller Family	Target Definition	Key Reported Endpoints
Ngan Kee (2007)	Obstetric anesthesia (cesarean under spinal)	Intermittent NIBP	On-off phenylephrine	SBP ±20% baseline	SBP time-in-range; hypotension incidence; MDAPE 6.0%
Sng/DIVA (2014)	Obstetric anesthesia (cesarean)	Continuous noninvasive AP (CNAP)	Rule-based dual-vasopressor (phenylephrine + ephedrine)	SBP maintenance near baseline	Hypotension incidence; BP precision; maternal symptom outcomes
Ngan Kee (2017)	Obstetric anesthesia (cesarean)	Intermittent NIBP	Proportional bolus vs. infusion strategy	SBP control around baseline	MDAPE 4.38–5.39%; wobble 3.5–4.2%; intervention burden
Sng/DIVA (2018)	Obstetric anesthesia (cesarean)	Continuous noninvasive AP (Nexfin)	Rule-based dual-vasopressor	SBP near baseline	Hypotension episodes; time-in-target; wobble/variability

Study (Year)	Clinical Setting	Sensor Modality	Controller Family	Target Definition	Key Reported Endpoints
Tan/ADIVA (2023)	Obstetric anesthesia (cesarean)	Continuous noninvasive AP (Nexfin)	Trend-based adaptive dual-vasopressor	SBP stabilization around obstetric target	Hypotension burden; MDAPE 13.1% vs. 9.5% DIVA (p=0.001); wobble
Joosten (2019)	Intraoperative moderate/high-risk noncardiac surgery (feasibility)	Invasive arterial line	PID + rule-based norepinephrine	MAP target range (tight control)	Time-in-target; hypotension burden; MDAPE 3–5%
Joosten (2021a)	Intraoperative major surgery (randomized comparison)	Invasive arterial line	PID + rule-based norepinephrine vs. manual	Clinician-defined MAP target range	Time-in-target; hypotension exposure; vasopressor dose patterns
Joosten (2021b)	Intraoperative individualized haemodynamic management	Invasive arterial line	Closed-loop NE integrated with fluid management logic	Individualized MAP maintenance target	Time-in-target; hypotension reduction; integrated haemodynamic performance
Kumar/CLAPS (2022)	Perioperative clinical validation cohort	Invasive arterial line	Multi-agent rule-based controller (4 vasoactives)	MAP target maintenance	Time-in-target 79.4% vs. 65.5% (p<0.001); MDAPE 9 vs. 15.5 (p=0.001); override frequency
Rinehart (2022)	Perioperative feasibility study	Invasive arterial line	PID + rule-based vasopressor control	SAP target (systolic strategy)	Time-in-target; hypotension burden; MDAPE
Desebbe (2022)	Post-cardiac surgery randomized trial	Invasive arterial line	PID + rule-based NE vs. nurse titration	MAP target range during postoperative stabilization	Time-in-target; hypotension burden; safety/feasibility
Coeckelenbergh (2024)	Postoperative high-risk	Invasive arterial line	PID + rule-based NE control	Tight MAP target window	Time-in-target; hypotension incidence;

Study (Year)	Clinical Setting	Sensor Modality	Controller Family	Target Definition	Key Reported Endpoints
	abdominal surgery				implementation feasibility

Abbreviations: AP, arterial pressure; CNAP, continuous noninvasive arterial pressure; MAP, mean arterial pressure; MDAPE, median absolute performance error; NIBP, noninvasive blood pressure; NE, norepinephrine; PID, proportional-integral-derivative; SAP, systolic arterial pressure; SBP, systolic blood pressure.

Note: *CLAPS, Closed-Loop Automated Blood Pressure Control System (Kumar 2022); DIVA, Double Intravenous Vasopressor Automated system (Sng/Sia group); ADIVA, Advanced DIVA (Tan 2023).*
